# Relationship between biomarkers of inflammation, oxidative stress and endothelial/microcirculatory function in successful aging versus healthy youth: a transversal study

**DOI:** 10.1186/s12877-015-0044-x

**Published:** 2015-04-08

**Authors:** Daniel Alexandre Bottino, Flávia Gomes Lopes, Francisco José de Oliveira, Anete de Souza Mecenas, Ruth Clapauch, Eliete Bouskela

**Affiliations:** Clinical and Experimental Research Laboratory on Vascular Biology (BioVasc), Biomedical Center, State University of Rio de Janeiro, Rio de Janeiro, RJ Brazil; UNATI, State University of Rio de Janeiro, Rio de Janeiro, RJ Brazil

**Keywords:** Microcirculation, Venous occlusion plethysmography, Nailfold videocapillaroscopy, Healthy elderly

## Abstract

**Background:**

There is a functional decline of endothelial- dependent vasodilatation in the aging process. The aims of this study were to investigate if various microcirculatory parameters could correlate to anthropometrical variables, oxidative stress and inflammatory biomarkers in successful aging and compare the results to young healthy controls.

**Methods:**

Healthy elderly women (HE, 74.0 ± 8.7 years, n = 11) and young controls (YC, 23.1 ± 3.6 years, n = 24) were evaluated through nailfold videocapillaroscopy (NVC), venous occlusion plethysmography (VOP) and laboratorial analysis. Functional capillary density (FCD) and diameters, maximum red blood cell velocity (RBCV_max_) during the reactive hyperemia response/RBCV_baseline_ after 1 min arterial occlusion at the finger base, time to reach RBCV_max_ were determined by NVC, peak increment of forearm blood flow (FBF) during the reactive hyperemia response (%Hyper) and after 0.4 mg sublingual nitroglycerin (%Nitro) by VOP and lipidogram, fibrinogen, fasting and postload glucose, oxidized LDL-cholesterol (oxLDL), sICAM, sVCAM, sE-Selectin, interleukines 1 and 6 and TNF-α by laboratorial analysis. Correlations and linear multiple regression (LMR) between %Hyper, %Nitro, microcirculatory parameters, oxidative stress and inflammatory biomarkers were investigated.

**Results:**

sVCAM, sE-Selectin and oxLDL were higher and RBCV_max_/RBCV_baseline_ and %Hyper lower in HE, while %Nitro and FCD remained unchanged. Fibrinogen, LDL-cholesterol, oxLDL correlated negatively to %Hyper while sVCAM correlated negatively to %Hyper and RBCV_max_/RBCV_baseline_. Healthy aged women presented dilated capillaries with sustained perfusion and endothelial dysfunction with preserved vascular smooth muscle reactivity. Fibrinogen, LDL-cholesterol, oxidized-LDL and sVCAM correlated negatively to endothelial function but not to microcirculatory parameters. Oxidized-LDL and sVCAM could determine %Hyper through LMR.

**Conclusion:**

Oxidized-LDL and sVCAM might be used as endothelial dysfunction biomarkers for elderly with normal cardiovascular risk factors.

## Background

Life span is increasing worldwide accompanied by high incidence and prevalence of arterial hypertension, diabetes mellitus and dyslipidemia that ultimately lead to atherosclerotic cardiovascular disease (CVD), the first mortality cause in many countries [1]. Endothelial dysfunction, expressed by reduced nitric oxide (NO) availability, is recognized as the crucial and earliest event for the onset of the atherosclerotic process.

Successful aging was first defined by Havighurst in 1961 as “the conditions of individual and social life under which the individual person gets a maximum of satisfaction and happiness” [2]. The concept that someone can “succeed” at aging involves diminishing the impact of diseases on the cardiovascular system and avoiding neoplasms. Besides, it is important to know the behavior of the cardiovascular system without disease in old age compared to young individuals because many pathophysiological conditions, mainly metabolic ones, will be more evident after 65 years and may bring frailty to many systems favoring the onset of diseases. For instance, it is believed that there is more production of reactive oxygen species and/or less antioxidant effect in the aging process which could cause oxidative stress and inflammation leading to an age-dependent endothelial dysfunction described in humans [3,4]. Adhesion molecules are pro-inflammatory proteins that play an important role in cell-cell or –matrix interactions during inflammation and immune response. Vascular cell adhesion molecule (VCAM) 1, endothelial selectin (E-selectin) and intercellular adhesion molecule (ICAM) 1, all control leukocyte adhesion to the endothelium [5].

Endothelial and microcirculatory functions can be assessed through different techniques, including venous occlusion plethysmography (VOP) and nailfold videocapillaroscopy (NVC). The main objectives of this study were to observe the influence of some anthropometrical parameters such as blood pressure and body mass index, oxidative stress (oxidized LDL-cholesterol), inflammatory biomarkers (sVCAM, sICAM, sE-Selectin, interleukines 1 and 6, TNF-α and C-reactive protein) and some classical cardiovascular risk factors (lipidogram, fibrinogen) to possible modifications of microcirculatory function and forearm blood flow using NVC and VOP, respectively of healthy elderly women compared to healthy young controls. We have hypothesized that inflammation and oxidative stress may be correlated to diminished endothelial function and impair structural and functional parameters in the microcirculation in the elderly.

## Methods

### Subjects

Thirty-five women were allocated into two groups: eleven healthy elderly (HE), over 65 years from the Geriatric Clinic (UNATI), and twenty-four young controls (YC), 18-30 years recruited among University students. Subjects were selected at the State University of Rio de Janeiro (UERJ) and considered to have successful aging if they had no cardiovascular risk factor except advanced age for the HE group, nor previous cardiovascular events. All subjects signed the written Informed Consent Form enclosed in the protocol approved by the Hospital Ethics Committee from the State University of Rio de Janeiro, in accordance with the Helsinki Declaration.

Main exclusion criteria involved cognitive impairment, frailty, heart disease, diabetes mellitus, glucose intolerance, smoking, high blood pressure, renal or autoimmune diseases and current hormone therapy (hormone replacement or oral contraceptives for young women).

Subjects visited the research laboratory on three days. First day: the protocol was explained and agreement from the patient obtained, followed by anamnesis and physical exam. Second day: Blood sample collection, after 12 hours fasting, measuring glucose, insulin, lipid profile and hSC-reactive protein and special inflammatory biomarkers, determined by ELISA: interleukines 1 beta [IL-1] and 6 [IL-6, High Sensitivity], TNF-α, soluble vascular cell adhesion molecule [sVCAM], soluble intercellular adhesion molecule [sICAM], soluble endothelial selectin [sE-Selectin], as well as a marker for oxidative stress (oxidized LDL). In addition, an oral glucose tolerance test was performed. Third day: patients were examined after 6 hours fasting using nailfold videocapillaroscopy (NVC) followed by venous occlusion plethysmography (VOP), as described below.

### Laboratory analysis

Total cholesterol, triglycerides and high density lipoprotein (HDL-cholesterol) were measured by enzymatic-colorimetric oxidase-peroxidase (reference value <240 mg/dl), enzymatic GPO-PAP (reference value <150 mg/dl) and enzymatic GPO-PAP (reference value >40 mg/dl), respectively. Low density lipoprotein (LDL-cholesterol) was calculated using Friedewald’s equation [6], (reference value < 130 mg/dl). Glucose and insulin levels were measured by enzymatic-colorimetric without pretreatment (reference value <99 mg/dl) and automated chemoluminescent methods (reference value <13 μUI/ml), respectively. Homeostasis model assessment of insulin resistance (HOMA-IR) index was calculated as fasting insulin (mU/l) x fasting glucose/22.5 (reference value 2.1 ± 0.7) [7]. High-sensitivity C-reactive protein determination was obtained by nephelometry (reference value <0.5 for inflammatory process and <0.11 for cardiovascular risk assessment) and fibrinogen by coagulometric technique (reference values between 200 and 400 mg/dl).

The levels of oxidized low density lipoprotein (oxidized LDL) were determined by spectrophotometry (reference value <0.5 nmol/mg ApoPt). Biomarkers such as TNF-α, IL-6, IL-1, E-selectin, ICAM-1, VCAM-1 and Oxidized LDL were evaluated using commercially available ELISA (Enzyme Linked Immunosorbent Assay) kits. Quantification of TNF-α and IL-6 was obtained using high sensitivity kits. Regarding kit sensitivity, the values were: sVCAM-1 = 0.17-1.26 ng/ml (mean = 0.6 ng/ml); sICAM-1 = 0.049-0.254 ng/ml (mean = 0.096 ng/ml); sE-selectin = 0.003-0.027 ng/ml (mean = 0.009 ng/ml); LDL-Oxidized = <1 mU/L; Il-6 = 0.016-0.110 pg/ml (mean = 0.039 pg/ml); TNF-α = 0.038-0.191 pg/ml (mean = 0.106 pg/ml) and IL-1 = 0.023-0.0140 pg/ml (mean = 0.057 pg/ml).

### Nailfold videocapillaroscopy

Subjects were seated on a high-based chair with the left upper arm raised at the heart level while the fourth finger was supported on an acrylic base, 2 cm above the palm level, mounted on the x–y stage of a three-eyepiece Leica DM/LM microscope (Wetzlar, Germany), equipped with an epiillumination system (100 W Xenon lamp). They waited for 30 minutes before the exam, in a temperature controlled room (24 ± 1ºC), for acclimatization. The fingertip was fixed to the acrylic base by a metal loop to minimize movements. The skin temperature of the finger was kept 24 ± 1ºC and monitored throughout the exam with an YSI Precision 4000A digital thermometer (Dayton, OH, USA) with the thermistor probe taped within 1 cm proximal to the nailfold. A pressure cuff (1 cm wide) was placed around the proximal phalanx and connected to a mercury manometer and a drop of mineral oil was spread over the observation site to improve image quality. Microcirculatory images were recorded and saved on DVDs within a closed circuit TV system.

Functional capillary density (FCD), considered an indicator of perfusion, was determined as the number of capillaries/mm^2^ with flowing red blood cells determined by the observation of their movement during all the experiment (magnification: 250x). Afferent (AF), apical (AP) and efferent (EF) capillary diameters and baseline red blood cell velocity (RBCV) were measured (magnification: 680x). All variables were determined in three contiguous microscopic regions, and the mean calculated to diminish variability. After 1-minute ischemia, with cuff (placed at the finger base) pressure above subject’s systolic blood pressure, the maximum value of RBCV during the reactive hyperemia response (RBCV_max_) and time (TRBCV_max_) taken to reach it were registered. The relation RBCV_max_/RBCV was determined to quantify vasodilatation compared to baseline condition. The analysis of all microcirculatory parameters was performed by CapImage software [8] by another member of the team, not aware of patients’ data. Inter-assay coefficient of variation from NVC ranged from 2.0% to 9.0% for measured parameters.

### Venous occlusion plethysmography

Subjects remained in a temperature controlled room (20-22°C) at supine position. Forearm blood flow (FBF), in ml/min/100 ml tissue, was measured using VOP (Hokanson, EC6, D.E., Bellevue, WA, USA) described elsewhere [9] in the non-dominant forearm, kept above the heart level, with a mercury-in-silastic strain gauge [10] placed on the upper third of the forearm at its maximum circumference. Briefly, the technique consists of brief interruption of venous drainage from the arm keeping arterial inflow unaltered. In other words, blood can enter the forearm but not escape. Such maneuver results in linear increase in forearm volume over time, proportional to arterial blood inflow, until venous pressure rises towards occluding pressure. One minute before FBF measurements, wrist pressure was set to 40 mmHg above systolic blood pressure to avoid hand shunt which makes arterial inflow nonlinear. Afterwards, there were inflating periods of 10 seconds using venous collecting pressure of 50 mmHg to avoid venous return followed by 5 seconds deflating periods in the arm, during two minutes. These periods were used for four measurements: baseline flow 1, reactive hyperemia response after 5 min forearm arterial occlusion, baseline flow 2, and flow 5 min after 0.4 mg sublingual nitroglycerin (Nitrolingual BurnsAdler Pharmaceuticals Inc, Charlotte, NC, USA), an endothelium-independent vasodilator. The percent increase in blood flow during the reactive hyperemia response was calculated in relation to baseline flow 1 (% HYPER), and its increase after nitroglycerin in relation to baseline flow 2 (% NITRO). There was a 20 min interval between the reactive hyperemia response and the second baseline flow measurement. The mean of the first 4 measurements in each recording period was used for analysis. Heart rate was measured continuously using lead II ECG. Before each phase, blood pressure was measured in the dominant arm.

### Statistical analysis

Results are presented as mean ± SD or as median [first-third quartiles] when non-normal distribution occurred. Data were analyzed with Statistica 8.0 software (Statsoft, Tulsa, Oklahoma, USA). Mann-Whitney U and Kruskal-Wallis tests were performed. Two analyses have been performed in sequence: first, Spearman’s test to compare each parameter (inflammatory, oxidative stress and biochemical biomarkers, microcirculatory parameters) to %HYPER OR %NITRO OR RBCVmax/RBCVbas. Second, the parameters who had statistically significant results in the first analysis were combined in many ways and studied through multiple linear regression where dependent variables were %HYPER OR %NITRO or RBCVmax/RBCVbas. P <0.05 was considered significant.

## Results

In order to assemble the healthy old women investigated in the present protocol, more than one hundred subjects were interviewed.

Table 1 shows clinical and laboratory data from young controls and healthy elderly. BMI, waist to hip ratio, and classical cardiovascular risk factors such as systolic pressure, fasting and post-load plasma glucose, triglycerides, total and non HDL cholesterol were all in the normal range although the HE group presented higher values compared to YC.

**Table 1:** **Subjects clinical and laboratorial data**

	**Young Controls (n = 24)**	**Healthy Elderly (n = 11)**	**p value**
Age	23.1 ± 3.6	74.0 ± 8.7*	**<0.001**
Body mass index (kg/m^2^)	21.7 ± 2.1	24.5 ± 3.9*	**0.002**
Waist to hip ratio	0.73 ± 0.04	0.83 ± 0.06*	**<0.001**
Systolic BP, mmHg	108 ± 11	128 ± 17*	**0.01**
Diastolic BP, mmHg	69 ± 8	69 ± 11	0.738
Heart Rate , bpm	65 ± 8	70 ± 10	0.650
Fasting glucose, mg/dl	81 ± 8	89 ± 4*	**<0.001**
Fasting insulin, μUI/ml	7 ± 3	6 ± 3	0.614
Postload Plasma Glucose, mg/dl	99 ± 20	125 ± 18*	**0.02**
HOMA-IR	1.44 ± 0.68	1.38 ± 0.62	0.729
Total cholesterol, mg/dl	172 ± 27	187 ± 30*	**0.01**
HDL cholesterol, mg/dl	68 ± 16	66 ± 17	0.717
LDL cholesterol, mg/dl	89 ± 17	109 ± 14*	**<0.01**
Non-HDL cholesterol, mg/dL	104 ± 17	129 ± 19*	**<0.01**
Triglycerides, mg/dl	67 ± 26	104 ± 37*	**<0.01**
Fibrinogen mg/dl	265 ± 50	340 ± 21*	**<0.01**

BP = blood pressure. *p <0.05 or p <0.01 related to Young Controls. Reference values: Fasting glucose <99 mg/dl; fasting insulin <13μUI/ml; Post load plasma glucose <140 mg/dl; Total cholesterol <240 mg/dl; triglycerides <150 mg/dl; high density lipoprotein (HDL-cholesterol) >40 mg/dl), respectively. Low density lipoprotein (LDL-cholesterol) was calculated using Friedewald’s equation^17^ using as reference value <130 mg/dl. Glucose and insulin levels were measured by enzymatic-colorimetric without pretreatment (reference value <99 mg/dl) and automated chemiluminescent methods (reference value <13μUI/ml), respectively. Homeostasis model assessment of insulin resistance (HOMA-IR) index was calculated as fasting insulin (mU/l) x fasting glucose/22.5 (reference value 2.1 ± 0.7)^24^. High-sensitivity C-reactive protein determination was obtained by nephelometry method (reference value <0.5 for inflammatory process and < 0.11 for cardiovascular risk assessment). Fibrinogen was determined by coagulometric method (reference values between 200 and 400 mg/dl).

Significant differences in LDL cholesterol and fibrinogen (Table 1), sVCAM, sE-Selectin and oxidized-LDL were observed between groups (Table 2), with HE presenting increased levels compared to young controls. sICAM, IL-1 beta, IL-6 high sensitivity, TNF-α and C-reactive protein were not different between groups.

**Table 2:** **Inflammatory and oxidative stress biomarkers**

	**Young Controls (n = 24)**	**Healthy Elderly (n = 11)**	
sVCAM (ng/ml)	19.3 [16.7-21.9]	29,7 [25-35]*	**0.017**
sICAM-1 (ng/ml)	9.8 [9.3-11.2]	11.5 [9.7-13.6]	0.057
sE-SELECTIN (ng/ml)	2.2 [1.1-3.1]	2.6 [2.4-3.4]*	**0.042**
Oxidized – LDL (mU/l)	35449 [31138-41238]	43909 [42154-47075]*	**0.015**
IL-1 beta (pg/ml)	0.4 [0.2-0.7]	0.3 [0.1-0.6]	0.515
IL-6 HS (pg/ml)	2.2 [1.5-5.4]	2.2 [1.1-2.9]	0.879
TNF-alpha (pg/ml)	1.8 [1.5-3.3]	1.8 [1.38-2.40]	1.000
hS C-reactive protein, mg/dl (mg/dl)	0.30 [0.07-0.59]	0.44 [0.23-0.63]	0.584

sVCAM= soluble Vascular Cell Adhesion Molecule; sICAM= soluble Intercellular Adhesion Molecule; sE-SELECTIN = soluble endothelial selectin; IL-1 beta = Interleukin-1 beta; IL-6 HS = Interleukin-6 High Sensitivity; TNF-alpha = Tumor Necrosis Factor–alpha. *p < 0.05 related to Young Controls.

**Figure 1:**
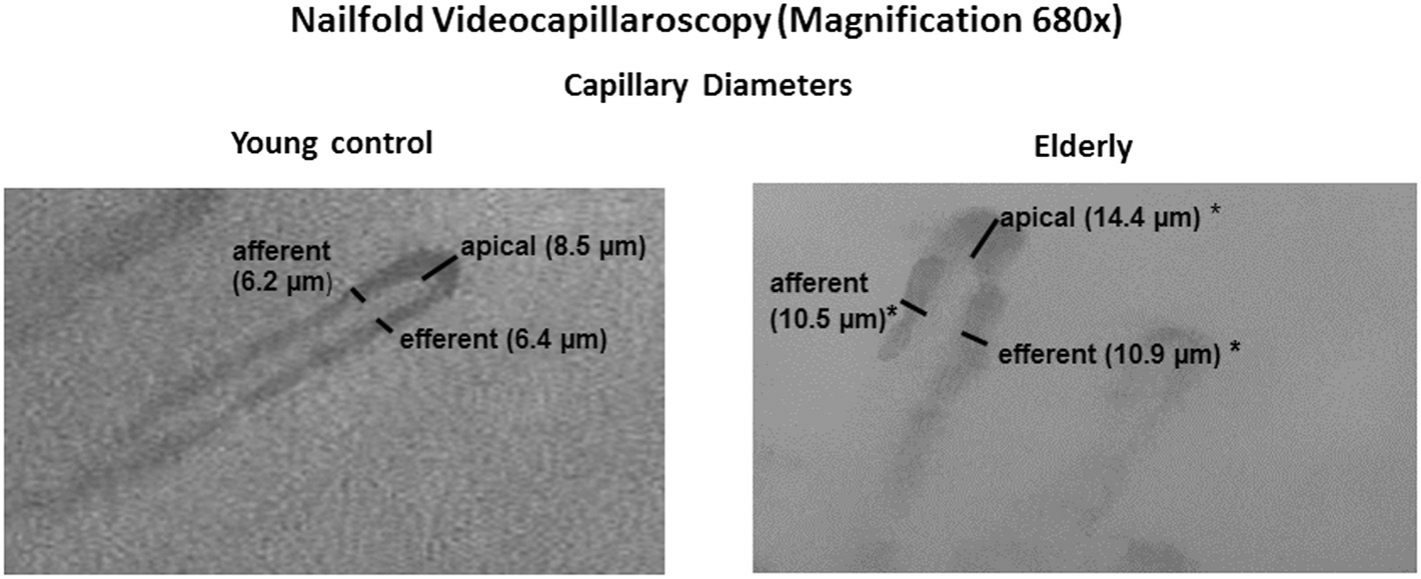
**Capillary diameters seen on Nailfold Videocapillaroscopy in the elderly and young controls groups.** Note that old people with healthy aging present larger capillaries than young controls (*p < 0.05).

Results from microcirculatory (NVC) and vasoreactivity analyses (VOP) are seen on Table 3. Microcirculatory morphological study by NVC showed enlarged capillaries in all viewed portions (apical, efferent and afferent) in HE compared to YC, capillary diameters in the elderly being on average 70% larger than in young persons (Figure 1). Regarding vascular function, we found no differences in FCD or on time to reach RBCV_max_ between groups. FCD in the elderly group represented 94% of FCD in young controls. However, the ratio RBCV_max_/RBCV_bas_ was decreased in HE compared to YC (1.23 [1.17-1.26] vs 1.35 [1.21-1.79], p < 0.05).

**Table 3:** **Microcirculatory evaluation (NVC) and vasoreactivity analysis (VOP)**

	**Young Controls (n = 24)**	**Healthy Elderly (n = 11)**	**p value**
**NVC** FCD (number of capillaries/mm^2^)	13.6 [12.6-14.9]	12.8 [11.9-13.7]	0.100
AFD (μm)	8.5 [6.1-11.2]	15.4 [13.7-16.3]*	**< 0.01**
APD (μm)	8.7 [7.1-10.7]	15.3 [13.6-17.0]*	**< 0.01**
EFD (μm)	8.7 [7.2-12.0]	14.9 [14.3-16.2]*	**< 0.01**
RBCVbasal	0.24 [0.22-0.28]	0.29 [0.29-0.30]*	**< 0.05**
RBCV_max_	0.35 [0.33-0.39]	0.35 [0.34-0.36]	0.616
RBCV_max_/RBCV_bas_	1.35 [1.21-1.79]	1.23 [1.17-1.26]*	**< 0.05**
Time to reach RBCV_max_/RBCV_bas_ (s)	5 [4-6]	4 [4-6]	0.408
**VOP**			
% Hyper	606 [508-744]	316 [256-465]*	**< 0.001**
% Nitro	208 [122-257]	187 [130-202]	0.530

FCD = Functional Capillary Density; Afferent, Apical and efferent capillary diameters (AFD,APD and EFD); RBCVmax/RBCVbas = increment of maximum red blood cell velocity (RBCV_max_) related to RBCV at baseline; % Hyper = percent of increment of forearm blood flow during reactive hyperemia related to baseline; % Nitro = increment of forearm blood flow after sublingual spray of nitroglycerine related to baseline. *p < 0.05 or p < 0.01 related to young controls.

Similarly, the vasodilatatory response during VOP reactive hyperemia was impaired in the HE group (%Hyper, HE = 316 [256-465] vs YC = 606 [508-744], p < 0.001) but preserved after sublingual nitroglycerine (%Hyper, HE = 187[130-202] vs YC = 208[122-257], p = 0.530.

**Figure 2:**
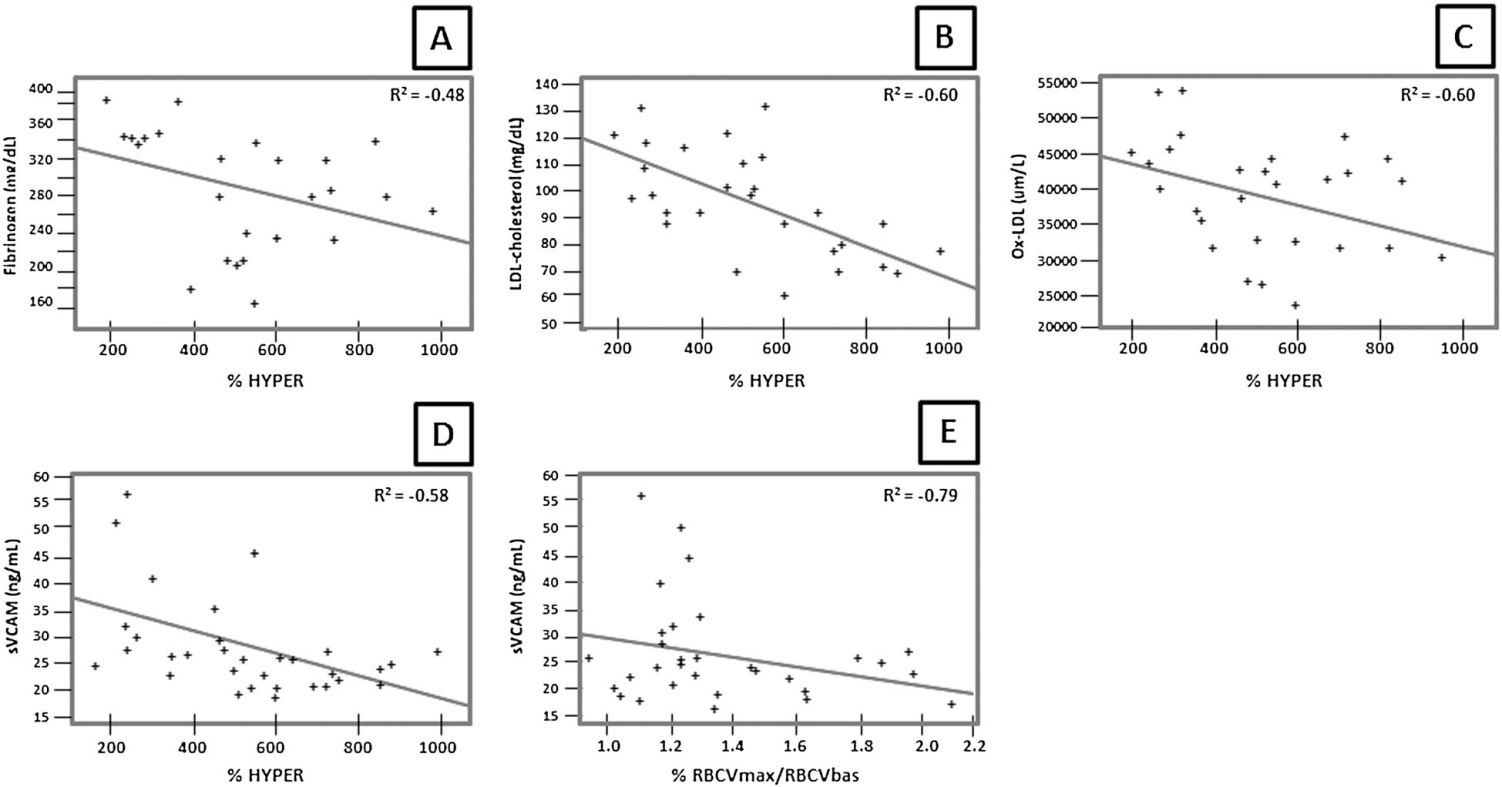
**Spearman correlations between fibrinogen (A), LDL-cholesterol (B), oxidized-LDL-cholesterol (C), sVCAM (soluble Vascular Adhesion Molecule) (D) and %Hyper (percent of increment of forearm blood flow during the reactive hyperemia related to baseline).** Box **E** shows Spearman correlation between sVCAM and RBCV_max_/RBCV_baseline_ (increment of maximum red blood cell velocity related to baseline values).

After the detection of impaired reactive hyperemia response with %Hyper, RBCVmax/RBCVbas and increased capillary diameters in the elderly, their correlation with altered anthropometrical and biochemical parameters in both groups was investigated, namely body mass index, waist to hip ratio, systolic blood pressure, fasting glucose, post load plasma glucose, total Cholesterol, LDL-cholesterol, fibrinogen, sVCAM, sE-Selectin and oxidized-LDL (Tables 1 and 2). There was a negative correlation between fibrinogen, LDL-cholesterol, oxidized-LDL and sVCAM with %Hyper (r = -0.48; r = -0.60; r = -0.60, r = -0.58, respectively). A negative correlation was also observed between sVCAM and RBCV_max_/RBCV_bas_ (r = -0.79) as well (Figure 2). No correlation was found between anthropometrical parameters and fasting glucose, post load plasma glucose, sVCAM, sE-Selectin, oxLDL, fibrinogen and LDL-cholesterol compared to capillary diameters.

Linear multiple regression analysis showed inverse correlations between %HYPER (dependent variable) and oxidized-LDL and sVCAM (independent ones). The intercept value was 11.5 with sVCAM beta = - 0.33 and oxidized-LDL beta = -0.34. Correlation result was 0.24 with p = 0.004.

## Discussion

New findings of this investigation in healthy aged women include the observation of dilated capillaries with preserved capillary density and higher levels of fibrinogen, LDL-cholesterol, oxidized-LDL and sVCAM correlated to impaired endothelium-dependent vasodilatation and not to microcirculatory parameters.

We have chosen extreme age-ranges, very young persons (23.1 ± 3.6 years) and old ones (74.0 ± 8.7 years), to characterize microvascular and endothelial functions and biomarkers alterations in the elderly without any drug intervention or hormone replacement therapy. Furthermore the use of biomarkers to demonstrate endothelial dysfunction, even in asymptomatic patients, would be useful to identify the most fragile ones concerning endothelial function.

Although few variables such as BMI, systolic blood pressure, glucose, LDL-cholesterol, triglycerides, fibrinogen presented statistical differences compared to YC group, in both groups classical cardiovascular risk factors were in the normal reference range. The biological aging process was studied in China by Bai and co-workers and among 208 physical, morphological, physiological and biochemical variables, only eight were chosen as candidate biomarkers of aging, which included echocardiographic parameters, intima-media thickness (IMT) and fibrinogen, demonstrating its importance on aging evolution [11].

Oxidative stress, analyzed through oxidized LDL, was increased in old healthy women in our study, as well as sVCAM and sE-selectin, inflammatory biomarkers. According to these results, we may speculate that aged healthy humans without any apparent cardiovascular risk factor may sustain an increased oxidative stress and low grade inflammatory conditions. The role of oxidative stress during aging is still a controversial topic. For example, impairment of e-NOS-dependent reactivity was demonstrated in cerebral arterioles of old rats [12,13]. However, Csiszar and co-workers could not prove longevity to be related to low cellular reactive species generation in fibroblasts from five great apes, including humans [14]. On the other hand, Rodriguez-Mañas and co-workers proposed that aged subjects produce enough nitric oxide but its vascular relaxation effects is counteracted by cyclooxygenase derived vasoconstricting factors and reactive oxygen species, resulting in decreased vasoreactivity [15]. We have found increased levels of an oxidative stress biomarker (oxidized-LDL) and of an inflammatory biomarker (sVCAM) to be inversely correlated to %Hyper.

Although oxidized LDL was increased in our study, it was not correlated to sVCAM or sE-Selectin. Other inflammatory biomarkers such as interleukins 1 and 6, TNF-α and C-reactive protein were not significantly different in HE and YC groups. Different results relating advanced age and inflammatory biomarkers are described in the literature. For instance, human population studies have associated interleukin-6, C-reactive protein and TNF-α to age-related chronic diseases, like coronary heart disease and disability [16-18]. Our old persons were healthy which may explain our results. On the other hand, Harris and co-workers have associated mortality to high circulating levels of IL-6 and C-reactive protein in healthy older persons [19]. Why healthy elderly have increased soluble adhesion molecules and oxidized-LDL remains to be elucidated.

Inflammation, oxidative stress and consequent endothelial dysfunction are common findings present in healthy aging process. These features are also present in diseases that most often occur in elderly persons like Alzheimer’s disease or cancer. For example, recent reports indicate that oxidative stress-mediated binding of advanced glycation endproduct (AGE) to its receptor (RAGE) can adversely increase the microglial inflammation and cytokines release [20]. Gaman et al. demonstrated that use of antioxidants decreased infectious complications in chronic lymphocytic leukemia in patients with median age of 65 years [21]. Around 1-2% of aged persons suffer from major depressive disorder also related to accumulation of cytokines (Interleukines 6 and 8). It should be mentioned that systemic inflammation interferes with serotonin metabolism reducing synaptic plasticity and hippocampal neurogenesis [22]. Thus inflammation, oxidative stress and endothelial dysfunction in healthy aging may cause physiological vulnerabilities favoring the appearance of new diseases. Furthermore, antioxidants and anti-inflammatory agents might be used as new targets of therapy to avoid or minimize the severity of diseases from aging.

Morphological microcirculatory analysis has shown dilated capillaries in older women in spite of the preservation of tissue perfusion, as FCD was similar in YC and HE groups. However, Russell and co-workers have reported that the degree of tortuosity and capillary branching and diameters were not different in old and young rats [23]. There was no correlation between anthropometrical and biochemical parameters versus microcirculatory structure in our group of old persons. We may speculate that aging is associated to deterioration of rheological blood behavior which could explain the dilated capillaries not found by Russell and co-workers. Indeed, Vayá and co-workers indicated that aging itself could affect hemorrheological parameters as there were increased blood and plasma viscosities, erythrocyte aggregation, fibrinogen and plasmatic lipids in old persons [24]. The increase in blood viscosity with age, independent of classic coronary heart disease risk factors, may suggest an effect of aging on red blood cells as pointed by Carallo and co-workers [25]. Sutera and co-workers, in 1985, have characterized the age-related changes in deformability of human erythrocytes showing decreased ability of older cells to change shape [26]. Salbas has also observed a 19% decrease in red blood cell deformability in elderly compared to young subjects [27]. Khecuriani and co-workers have also observed an excessive number of large-sized matured (aged) erythrocytes in the blood of old persons [28]. Besides deformability, Abe and co-workers described the presence of higher density erythrocytes with aging in rats which might be an extra factor to interfere into capillary structure [29].

The vasoreactivity analysis revealed lower RBCV_max_/RBCV_bas_ at NVC and %Hyper at VOP in the HE compared to YC, without statistical difference to %Nitro suggesting the existence of decreased endothelial nitric oxide (NO) bioavailability with conserved vascular smooth muscle dilating capacity. The reactive hyperemia response is a physiological stimulus for the release of NO (responsible for 30% of the response [30]), adenosine, endothelium-derived hyperpolarizing factor (EDHF) and prostaglandins. Endothelial function has been shown to decline with aging elsewhere [31] in agreement to our data.

Decreased number of endothelial cells, accumulation of advanced glycosylation end products in the vascular sub-endothelium causing oxidative stress and deficient concentration of L-arginine are described to reduce NO availability and consequently to produce lower endothelial-dependent vasodilatation [12,32]. In accordance with observed impaired vasodilatation, we have found increased oxidized-LDL levels, representing an oxidative stress profile.

Although endothelial dysfunction and the presence of cardiovascular risk factors are not novelty in the literature, further analysis was performed in order to verify which of the cardiovascular risk factors evaluated in this study would be correlated to %Hyper or RBCV_max_/RBCV_bas_. A negative correlation was detected between LDL-cholesterol, fibrinogen, oxidized-cholesterol, sVCAM and %Hyper. sVCAM was highly correlated to RBCV_max_/RBCV_bas_ as well (r = -0.79) (Figure 2). Probably, LDL-cholesterol, fibrinogen, sVCAM and oxidized-LDL are significant contributors to age–related endothelial-dependent vasodilation impairment.

Fibrinogen and LDL-cholesterol have been demonstrated to be associated to endothelial dysfunction in the elderly, in our study these parameters were in the normal range and therefore not responsible for the detected endothelial dysfunction, but oxidized-LDL and sVCAM showed higher levels compared to young controls and high inverse correlation to %Hyper (r = -0.60 and -0.58, respectively). Linear multiple regression analysis has been performed and it was observed a dependence of %HYPER to oxidized-LDL and sVCAM with statistical significance (p <0.05). Thus, we may speculate that oxidized-LDL and sVCAM could be used in the future to infer endothelial dysfunction in this population when fibrinogen and LDL-cholesterol are in the normal range.

### Study limitation

The number of studied elderly persons is small but all of them have very low cardiovascular risk, normal biochemical parameters in a situation called successful aging. This kind of patient is rare because most aged persons have chronic diseases like Diabetes Mellitus, high blood pressure and other cardiovascular diseases. There is growing interest in studying the impact of aging itself on inflammatory and oxidative profiles related to endothelial function and we have found statistically significant results. Endothelial dysfunction, inflammation and oxidative stress in healthy aging may be treated to avoid the progression of atherosclerosis and cardiovascular diseases that will shorten the life span. This study was performed only with elderly women as the general hospital from the University receives daily 75% women and 25% men. Since it is difficult to find many healthy elderly, we have decided to choose only women.

Oxidative stress was assessed only by lipid peroxidation. In future studies, the use of other biomarkers together with GSSG/GSH ratio or the evaluation of the reactive oxygen species should be considered to improve the understanding of the oxidative stress profile.

## Conclusions

Our results have shown that aged healthy women presented (1) dilated capillaries with preserved tissue perfusion without correlation to anthropometrical and biochemical parameters; (2) Impaired endothelium-dependent vasodilation inversely correlated to fibrinogen, LDL-cholesterol, oxidized-LDL and sVCAM which could be related to pro-coagulant, oxidative stress and inflammatory profile, independent of pathologies. Oxidized-LDL and sVCAM could determine %HYPER through Linear Multiple Regression. Fibrinogen and LDL cholesterol complemented by oxidized-LDL, as well as sVCAM, might be used as biomarkers to determine the severity of endothelial dysfunction in successful aging.
